# Clinical and sociodemographic predictors of inpatient admission after presentation in a psychiatric emergency room: an observational study

**DOI:** 10.1186/s13033-023-00618-2

**Published:** 2023-12-05

**Authors:** Gianna L. Gisy, Sermin Toto, Stefan Bleich, Hannah B. Maier, Johanna Seifert

**Affiliations:** Department of Psychiatry, Social Psychiatry and Psychotherapy, Hannover Medical School, Carl-Neuberg-Straße 1, 30625 Hannover, Germany

**Keywords:** Emergency psychiatry, Admission predictor, Discharge, Suicide, Suicide attempt, Self-harm, Mental Illness, Delusions, Frequent visitors

## Abstract

**Background:**

The admission decision after presentation in the psychiatric emergency room (PER) has potentially far-reaching consequences for the patient and the community. In a short amount of time, information must be collected and evaluated for a well-founded admission decision. The present study aimed to identify risk factors associated with inpatient psychiatric admission (IPA) after previous emergency presentation to the PER.

**Methods:**

Electronic patient records for all patients presenting in the PER of Hannover Medical School (MHH) in the year 2022 were retrospectively examined (n = 2580). Out of these, 2387 were included in this study. Two multivariate binary logistic regression analyses were performed to identify risk factors for IPA within sociodemographic, circumstantial and clinical variables.

**Results:**

1300 (54.5%) consultations resulted in IPA. The most significant sociodemographic and circumstantial risk factors for IPA were found to be suicide attempt (depending on method: OR 9.1–17.4), aggressive behavior towards people prior to presentation (OR 2.9, 95% CI 1.7–4.8), previous psychiatric treatment (OR 1.8, 95% CI 1.4–2.3) and transfer from another hospital or medical discipline of MHH as means of presentation (OR 6.3, 95% CI 3.0–13.0). Among psychopathological aspects, suicidal ideation (OR 3.8, 95% CI 2.9–4.9), suicidal intent (OR 116.0, 95% CI 15.9–844.8), disturbance of orientation (OR 3.7, 95% CI 2.5–5.3), delusions (OR 2.3, 95% CI 1.6–3.1), visual hallucinations (OR 2.9, 95% CI 1.6–5.1), hopelessness/despair (OR 2.4, 95% CI 1.7–3.2) and inhibition of drive (OR 1.6, 95% CI 1.3–2.1) were significantly associated with IPA.

**Conclusions:**

The admission decision is a complex process influenced by a multitude of sociodemographic, circumstantial and clinical factors. A deeper understanding of the decision-making process can be used to improve patient care and facilitate the evaluation process in the PER.

**Supplementary Information:**

The online version contains supplementary material available at 10.1186/s13033-023-00618-2.

## Background

Constituting about 7–9% of presentations, patients in need of psychiatric treatment represent a significant portion of consultations in the emergency room [1–3]. A psychiatric emergency is defined as “an acute disturbance in thought, behavior, mood, or social relationship, which requires immediate intervention as defined by the patient, family, or social unit” [4] by the American Psychiatric Association. While not all patients presenting in the psychiatric emergency room (PER) present with an actual psychiatric emergency, physicians of course still must assess the need for further treatment. Decisions including necessity of acute treatment, diagnostic measures and the need for immediate inpatient care have to be made in a short amount of time with only limited resources [5]. Due to increasing numbers of emergency psychiatric consultations [2, 6] resulting in less time per patient, quick decision-making is of immense importance.

During the decision process, in which the necessity for inpatient care is determined, physicians must also be aware of the potential consequences of the admission decision: Besides the economical consideration of optimal allocation of limited hospital beds, the admission decision also greatly impacts the course of further treatment and maybe even of the illness [7]. Unnecessary admission as well as premature release can both have severe adverse consequences affecting not only the patient but potentially family members and the community [8]. While premature release may result in violence against a third party, suicide or aggravation of the underlying illness, unnecessary admissions may lead to increasing costs, stigmatization, functional impairment and loss of jobs [8, 9].

Analyzing the factors associated with inpatient psychiatric admission (IPA) and discharge can improve the decision-making process in two ways: First, it can clarify which factors need to be examined more thoroughly since not all variables impact admission decisions in the same manner. Secondly, through the examination of admission behavior, unreasonable decision-making can be detected and improved upon.

Past research on variables influencing inpatient admission after emergency psychiatric treatment is largely outdated [7, 10–15] and from other countries such as Italy [16–18] and the USA [19–24] whereas data from Germany is limited [2, 25, 26]. The present study aims to assess which sociodemographic and circumstantial factors as well as aspects of the psychopathological assessment (PPA) influence subsequent inpatient psychiatric admission after presentation in the psychiatric emergency room of a large university hospital in Lower Saxony, Germany.

## Methods

### Setting

In Hannover and surrounding areas, Hannover Medical school (German: Medizinische Hochschule Hannover, MHH) provides service to about 138,500 inhabitants and represents one of four psychiatric hospitals [27]. Among these, the MHH has the only PER which also has on-site access to other disciplines such as internal medicine, neurology and gynecology that are also represented in the central emergency department. This fact, in addition to being the only psychiatric hospital within the city borders of Hannover, results in presentation of a large number of patients at the MHH that do not belong to MHH’s official catchment area and will later be transferred to their designated psychiatric hospital for further care.

Before medical treatment can take place in the central emergency department, patients are assessed by a specialized emergency department nurse who then assigns the patient to the appropriate medical specialty. Following this triage, further treatment is provided by the physician from the designated discipline. This includes decisions regarding diagnostic measures, acute treatment and the inpatient admission. In the PER, the primary diagnostic tool is the PPA, as well as a physical examination and laboratory tests. When deemed necessary, consultations by other medical disciplines may be requested. In some cases, especially when somatic issues are more acute and potentially life-threatening, the patient may be admitted to the inpatient care of other medical specialties.

### Collection of data

The data was collected by retrospectively analyzing the routinely compiled electronic reports generated in the PER. Of particular interest was information regarding sociodemographics (e.g., age, living arrangement), circumstantial factors (e.g., mode and day of presentation) and the PPA. The PPA was documented according to the “Arbeitsgemeinschaft für Methodik und Dokumentation in der Psychiatrie” (AMDP)-System [28], which is a tool used for a structured and standardized assessment in psychiatry and includes definitions for each of the variables. It is widely used in German-speaking countries. In the present study 62 variables of the PPA according to the AMDP-System were assessed.

Psychiatric diagnosis was recorded according to the International Classification of Disease 10th Version (ICD-10) [29] and classified into ten main diagnosis groups, as shown in Table 1. The diagnostic variable “others” contains diagnoses with small numbers of cases, e.g., eating disorders and attention deficit hyperactivity disorder. If a patient presents with an acute intoxication and addiction to alcohol, if no other outstanding diagnoses (e.g., schizophrenia) were present, the alcohol addiction was considered to be the main diagnosis.

**Table 1:** Primary diagnoses of the study population according to ICD-10

Diagnosis	All patients		IPA		Discharged		Chi-square test post hoc
	N	%	n	%	n	%	
None	5	0.2	0	0.0	5	0.5	0.014*
F01-03	46	1.9	31	2.4	15	1.4	0.075
F10	571	23.9	332	25.5	239	22.0	0.043*
F11-18	79	3.3	29	2.2	50	4.6	0.001*
F19	104	4.4	65	5.0	39	3.6	0.092
F20-23	333	14.0	217	16.7	116	10.7	< 0.001**
F30-31	87	3.6	59	4.5	28	2.6	0.011*
F32-33	405	17.0	235	18.1	170	15.6	0.114
F40-48	391	16.4	110	8.5	281	25.9	< 0.001**
F60-61	151	6.3	100	7.7	51	4.7	0.003*
Others	215	9.0	122	9.4	93	8.6	0.481
Total	2387	100.00	1300	54.50	1087	45.5	

ICD-10: International Classification of Disease 10th Version, IPA: inpatient psychiatric admission, n: number of consultations, others: F04-F09, F24-F29, F34, F50-F54, E51, G25, G31, G47, R40, R44-R45, R63, T39, T43, T88, U07, X84, Z59, Z73, Z91; * statistically significant, **highly statistically significant

“Suicide attempt (SA) prior to presentation” was evaluated using the definition of SA in the “S2k-Guidleline Emergency Psychiatry” [5]: “every action with non-lethal outcome, in which the individual either purposefully engages in a nonhabitual behavior that would cause self-harm in the absence of third-party intervention, or intentionally ingests a substance at a dose in excess of that prescribed or generally considered therapeutic, with the goal of effecting change through current or anticipated consequences”.

Similar to Seifert et al. [27], cases in which a psychopathological item appeared to be present but could not be verified with certainty, e.g., because of the patient being uncooperative, the item was classified as “being present”. If negated or no indication on the PPA-item could be found, it was not selected.

In case a patient from a different catchment area was transferred to their designated psychiatric hospital after presenting in MHH’s PER, the patient was included in the group of IPA.

### Selection of the study population

**Fig. 1:**
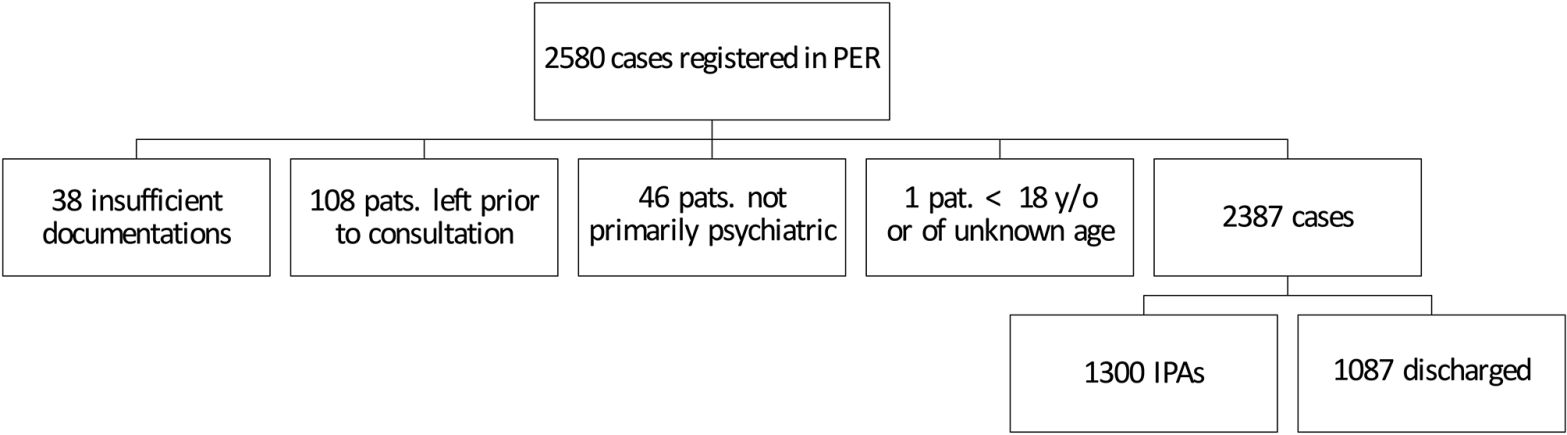
Selection of the study population. PER: psychiatric emergency room, pats.: patients, not primarily psychiatric: wrongly triaged or somatic main diagnosis or inpatient non-psychiatric admission, y/o: years old, IPA: inpatient psychiatric admission

All patients presenting in the PER in the year 2022 (01.01.2022–31.12.2022) were analyzed, consisting of 2580 cases and 1648 patients. As the whole year was included, no bias regarding season is expected [30]. If the electronic documentation was insufficient, a patient left before a thorough examination took place, did not present with a primarily psychiatric indication or was under 18 years old, the patient was excluded from further analysis, leaving 2387 consultations and 1517 patients (Fig. 1). Because the present study analyses factors associated with IPA and the decision for or against IPA must be reevaluated for each individual presentation as the circumstances and psychopathological symptoms of the patient may be different, the present study analyses each emergency presentation separately.

### Data analysis

Statistical analysis was performed using the program SPSS© version 28.

First, in order to compare patients admitted to inpatient psychiatric care to those who were discharged, a descriptive analysis for all variables using chi-square tests for nominal and categorical variables and T-tests for metric variables was performed. For categorical variables, an additional chi-square post hoc analysis was performed, if the initial chi-square test of the variable had more than one degree of freedom and reached a significance level *p < 0.05*. The frequency of “frequent visitors”, defined as patients presenting at least four times during the study period [31], was calculated. Because of the primary exploratory nature of this first descriptive analysis, we did not adjust for multiple testing.

For the pre-selection of variables entering the multivariate logistic regression analyses, all variables were analyzed using univariate logistic regression. Only variables that showed a *p* − *Value <* 0.25 in the univariate testing were included in the multivariate logistic regression analysis [32]. This rather loose inclusion criteria of *p <* 0.25 is justified by the goal of identifying potential predictors rather than testing a hypothesis [33]. Aspects of the PPA were only included if they were documented for in at least 50 cases. The multivariate regression analysis was adjusted for age and gender by including these variables in a separate step, regardless of their level of significance. The main diagnosis was excluded from multivariate logistic regression analysis due to a high level of instability of diagnoses made in PER [34, 35]. Further, as aspects of PPA are expected to show a high correlation with certain diagnoses (e.g., delusions and schizophrenia), psychiatric diagnoses are most likely not independent predictors of IPA.

Two separate multivariate logistic regressions were performed using backwards elimination with the inclusion criteria *p* = 0.05. The first analysis included only sociodemographic (e.g., age, gender) and setting variables, which are objective and mostly already known to the physician before the examination takes place (e.g., referral by ambulance). The second multivariate logistic regression analysis contains all variables (”overall analysis”). A significance level of *p* = 0.05 (two-sided) was set as threshold for statistical significance. Due to the high number of significant findings and in order to focus on the most relevant predictors, only findings with a *p* < 0.001 are presented in the following discussion.

### Ethical approval

The Clinical Ethics Committee of Hannover Medical School issued the ethical approval for this study (No 10740_BO_K_2023). This study adheres to the Declaration of Helsinki and its later amendments.

## Results

Out of 2580 cases presented during the one-year study period, 2387 cases were analyzed, averaging 6.54 ± SD 2.67 presentations per day. 1517 individual patients accounted for the 2387 consultations. Therefore, the average patient utilization of the PER was 1.57 ± SD 1.83 (median 1, range 1–24). The 15 patients to present most frequently in the PER (i.e., 1% of all patients) were responsible for 10% of the emergency presentations. A total of 93 frequent visitors (i.e., 6.1% of all patients) accounted for 27.5% of emergency presentations. 1300 consultations (54.5%) resulted in IPA, whereas 1087 patients (45.5%) were discharged.

### Sociodemographic and circumstantial characteristics

Basic sociodemographic and circumstantial characteristics of the study population are shown in Table 2. In 53.2% of the cases, the patient was male, followed by 45.3% women, while 1.6% identified as “transgender”. The age ranged between 18 and 95 years (median 40 years, mean 42.9 years, SD 17.6 years).

**Table 2:** Sociodemographic and circumstantial characteristics of the study population

	All Patients		IPA		Discharged		Chi-square test		Chi-square test post hoc	
	N = 2387	%	n = 1300	%	n = 1087	%	df	*p*-Value	df	*p*-Value
**Gender**										
Female	1081	45.3	596	45.8	485	44.6	2	0.107	1	0.548
Male	1269	53.2	678	52.2	591	54.4			1	0.28
Transgender	37	1.6	26	2.0	11	1.0			1	0.052
**Means of presentation**										
Walk-in	1247	52.2	571	43.9	676	62.2	3	< 0.001**	1	< 0.001**
By ambulance	817	34.2	481	37.0	336	30.9			1	0.002*
By police escort	232	9.7	168	12.9	64	5.9			1	< 0.001**
Transfer	91	3.8	80	6.2	11	1.0			1	< 0.001**
Accompanied	421	17.6	226	17.4	195	17.9	1	0.723		
**Previous psychiatric treatment**										
Yes	1670	70.0	966	74.3	704	64.8	1	< 0.001**		
**Aggressive prior to presentation**										
Aggressive in general	204	8.5	155	11.9	49	4.5	1	< 0.001**		
Aggressive towards people	155	6.5	122	9.4	33	3.0	1	< 0.001**		
Aggressive towards objects	59	2.5	45	3.5	14	1.3	1	< 0.001**		
**Suicide attempt prior to presentation**										
Yes	101	4.2	96	7.4	5	0.5	1	< 0.001**		
**Intoxication**										
None	1669	69.9	880	67.7	789	72.6	3	0.032*	1	0.009*
Alcohol	549	23.0	313	24.1	236	21.7			1	0.171
Non-alcohol drugs	100	4.2	63	4.8	37	3.4			1	0.080
Both	69	2.9	44	3.4	25	2.3			1	0.115
**Coersive measures**										
Mechanical restraint	71	3.0	60	4.6	11	1.0	1	< 0.001**		
**Living arrangement**										
With others	656	27.5	319	24.5	337	31.0	6	< 0.001**		< 0.001**
Homeless	170	7.1	88	6.8	82	7.5			1	0.464
Alone	555	23.3	347	26.7	208	19.1			1	< 0.001**
Psychiatric residency/nursing home	315	13.2	186	14.3	129	11.9			1	0.079
Refugee shelter	21	0.9	14	1.1	7	0.6			1	0.259
Others	10	0.4	6	0.5	4	0.4			1	0.725
Missing information	660	27.6	340	26.2	320	29.4			1	0.074
**Work status**										
(Self-)employed	433	18.1	198	15.2	235	21.6	5	< 0.001**	1	< 0.001**
Without work	393	16.5	209	16.1	184	16.9			1	0.577
Student	137	5.7	66	5.1	71	6.5			1	0.128
Sheltered workshop	14	0.6	7	0.5	7	0.6			1	0.737
Retired	294	12.3	205	15.8	89	8.2			1	< 0.001**
Missing information	1116	46.8	615	47.3	501	46.1			1	0.553

IPA: inpatient psychiatric admission, n: number of consultations, df: degrees of freedom, transfer: transfer from another hospital or another medical discipline within the MHH, homeless: without permanent home/homeless shelter, others: prison or women’s/men’s shelter; *statistically significant, **highly statistically significant

Most patients presented by walk-in (52.2%), about one third was brought by an ambulance and in about 10% of the cases the patient was escorted by police. Subsequent IPA was observed significantly more often in patients who arrived by ambulance (37.0% vs. 30.9%, *p* = 0.002), police escort (12.9% vs. 5.9%, *p <* 0.001) or as a transfer (6.2% vs. 1.0%, *p <* 0.001). 17.6% of the patients were accompanied by others (e.g., caregivers, parents, spouses).

Regarding living arrangements, most patients lived with others (e.g., family, partner, flat sharing; 27.5% of all patients) or alone (23.3%). 13.2% lived in a nursing home or a psychiatric residency. About 7.1% did not have a permanent residence. It should be noted that in a fair number of cases (27.6%) the information on the current living arrangement was not available. Patients living alone were significantly more often admitted to inpatient psychiatric care (26.7% vs. 19.1%, *p <* 0.001) than those living with others (24.5% vs. 31.0%, *p <* 0.001).

In terms of employment status, most cases were fairly evenly distributed between being (self-) employed (18.1% of all patients), unemployed (16.5%) and retired (12.3%), although information on employment status was not available in almost half of the cases. Retired patients were admitted significantly more often (15.8% vs. 8.2%, *p <* 0.001), while the contrary applied for (self-) employed patients (15.2% vs. 21.6%, *p <* 0.001).

In 8.5% of the consultations, patients had presented aggressive behavior beforehand. Patients with previous aggressive behavior were significantly more likely to be admitted to inpatient psychiatric care than discharged (11.9% vs. 4.5%, *p <* 0.001). Coercive measures in form of mechanical restraints were observed more than four-times as often in the IPA group (4.6% vs. 1.0%, *p <* 0.001). 4.2% of emergency presentations constituted patients with a SA prior to presentation, out of which a significant majority was admitted to inpatient psychiatric care (7.4% vs. 0.5%, *p <* 0.001).

### Distribution of primary psychiatric diagnosis

Table 1 shows the distribution of primary diagnoses in the PER. Alcohol-related disorders (F10) were the most frequent primary diagnosis (23.9%), followed by depressive disorders (F32-F33; 17.0%), neurotic, stress-related and somatoform disorders (F40-48; 16.4%) and schizophrenia, schizotypal and delusional disorders (F20-F23; 14.0%).

For most diagnoses a significant difference was found between the IPA group and the discharged patients. Among patients with a primary diagnosis of alcohol-related disorders, significantly more were admitted to inpatient psychiatric care than discharged (25.5% vs. 22.0%, *p* = 0.043). Patients suffering from a primary diagnosis of schizophrenia (F20-23) were also found significantly more often in the IPA group than in the discharged group (16.7% vs. 10.7%, *p <* 0.001). The same applies for patients diagnosed with mania/bipolar disorder (F30-F31), who were more likely to be admitted than discharged (4.5% vs. 2.6%, *p* = 0.011). On the other hand, patients with diagnosis related to single drug intoxication/abuse of drugs other than alcohol (F11-18) or neurotic, stress-related and somatoform disorders (F40-48) were discharged significantly more often (2.2% vs. 4.6%, *p* = 0.001 and 8.5% vs. 25.9%, *p <* 0.001).

### Psychopathological characteristics

The most common elements of the PPA observed in patients in the PER where depressed mood (66.2%), disturbed apperception/concentration (56.6%) and fears (50.3%), followed by lack of drive (46.1%), circadian disturbances/disturbance of sleep and vigilance (31.0%) and motor restlessness/tenseness (27.9%), as can be seen in Table 3. Disturbances of consciousness, orientation, apperception/concentration and memory were observed significantly more frequently in the IPA group than in the discharged group (each *p <* 0.001). Among the formal thought disorders, patients in the IPA group were more likely to present with disruption of thought (2.5% vs. 0.8%, *p* = 0.004), incoherence (10.2% vs. 3.3%, *p <* 0.001) and accelerated thinking (6.5% vs. 2.6%, *p <* 0.001) than those who were discharged.

**Table 3:** Selected aspects of the psychopathological assessment (PPA) as found in the study population

	All Patients		IPA		Discharged		Chi-square test		Chi-square test post hoc	
	N = 2387	%	n = 1300	%	n = 1087	%	df	*p*-Value	df	*p*-Value
**Physical appearance**										
Unkempt/inappropriate attire	370	15.5	257	19.8	113	10.4	1	< 0.001**		
**Disorders of consciousness, orientation, attention and memory**										
Disturbance of consciousness	150	6.3	110	8.5	40	3.7	1	< 0.001**		
Any disturbance of orientation	301	12.6	242	18.6	59	5.4	1	< 0.001**		
Disturbed apperception/concentration	1352	56.6	826	63.5	526	48.4	1	< 0.001**		
Disturbed memory	520	21.8	338	26.0	182	16.7	1	< 0.001**		
**Formal thought disorders**										
Inhibited/retarded thinking	446	18.7	291	22.4	155	14.3	1	< 0.001**		
Accelerated thinking	112	4.7	84	6.5	28	2.6	1	< 0.001**		
Rumination	470	19.7	237	18.2	233	21.4	1	0.050		
Rambling	135	5.7	85	6.5	50	4.6	1	0.042*		
Tangential thinking	119	5.0	85	6.5	34	3.1	1	< 0.001**		
Thought blocking/disruption of thought	41	1.7	32	2.5	9	0.8	1	0.004*		
Incoherence/derailment	168	7.0	132	10.2	36	3.3	1	< 0.001**		
**Delusions**										
Delusions in general	393	16.5	294	22.6	99	9.1	1	< 0.001**		
Delusions of reference	32	1.3	26	2.0	6	0.6	1	0.002*		
Delusions of persecution/impairment	275	11.5	210	16.2	65	6.0	1	< 0.001**		
**Disorders of perception**										
Auditory hallucinations in general	274	11.5	206	15.8	68	6.3	1	< 0.001**		
Visual hallucinations	125	5.2	101	7.8	24	2.2	1	< 0.001**		
**Ego (boundary) disturbances**										
Any ego disturbance	277	11.6	181	13.9	96	8.8	1	< 0.001**		
**Worries and compulsion**										
Fears	1200	50.3	648	49.8	552	50.8	1	0.649		
Compulsions	87	3.6	50	3.8	37	3.4	1	0.566		
**Disturbances of affect**										
Perplexity	41	1.7	31	2.4	10	0.9	1	0.006*		
Feeling of loss of feeling	69	2.9	45	3.5	24	2.2	1	0.069		
Depressed mood	1580	66.2	852	65.5	728	67.0	1	0.461		
Hopelessness/despair	380	15.9	280	21.5	100	9.2	1	< 0.001**		
Anxiety	108	4.5	69	5.3	39	3.6	1	0.044*		
Euphoria	28	1.2	21	1.6	7	0.6	1	0.028*		
Dysphoria/irritability	270	11.3	166	12.8	104	9.6	1	0.014*		
Inner restlessness	285	11.9	128	9.8	157	14.4	1	< 0.001**		
Parathymia (incongruent affect)	47	2.0	39	3.0	8	0.7	1	< 0.001**		
**Disorder of drive and psychomotor activity**										
Inhibition/lack of drive	1101	46.1	643	49.5	458	42.1	1	< 0.001**		
Increased drive	203	8.5	142	10.9	61	5.6	1	< 0.001**		
Motor restlessness/tenseness	665	27.9	450	34.6	215	19.8	1	< 0.001**		
Motor redardation	222	9.3	154	11.8	68	6.3	1	< 0.001**		
Mannerisms/histrionics	67	2.8	53	4.1	14	1.3	1	< 0.001**		
**Other disturbances**										
Aggressiveness	76	3.2	62	4.8	14	1.3	1	< 0.001**		
Self-harm	205	8.6	138	10.6	67	6.2	1	< 0.001**		
Lack of insight into illness	355	14.9	271	20.8	84	7.7	1	< 0.001**		
Circadian disturbances/disturbances of sleep and vigilance	740	31.0	387	29.8	353	32.5	1	0.155		
**Suicidal ideation**										
No suicidal ideation	1531	64.1	618	47.5	913	84.0	2	< 0.001**	1	< 0.001**
Suicidal ideation	746	31.3	591	45.5	155	14.3			1	< 0.001**
Missing information	110	4.6	91	7.0	19	1.7			1	< 0.001**
**Suicidal intent**										
Distance to suicidal intent	2005	84.0	931	71.6	1074	98.8	2	< 0.001**	1	< 0.001**
Lack of distance to suicidal intent	241	10.1	240	18.5	1	0.1			1	< 0.001**
Missing information	141	5.9	129	9.9	12	1.1			1	< 0.001**

IPA: inpatient psychiatric admission, n: number of consultations, df: degrees of freedom. * statistically significant, ** highly statistically significant

Delusions were present in 16.5% of the cases, which were detected significantly more often in the IPA group (22.6% vs. 9.1%, *p <* 0.001). Moreover, auditory and visual hallucinations showed a highly significant difference between admitted and discharged patients (each *p <* 0.001).

Among disturbances of affect, perplexity (2.4% vs. 0.9%, *p* = 0.006), hopelessness/despair (21.5% vs. 9.2%, *p <* 0.001) and parathymia (3.0% vs. 0.7%, *p <* 0.001) were documented significantly more often in the IPA group. Patients complaining of inner restlessness were less often admitted to inpatient psychiatric care (9.8% vs. 14.4%, *p <* 0.001). Aggressive behavior with at least verbal threats (4.8% vs. 1.3%, *p <* 0.001) and self-harm (10.6% vs. 6.2%, *p <* 0.001) were also significantly more common in the IPA group.

In 31.3% of cases, patients stated suicidal ideations, which were reported significantly more often from patients admitted to inpatient psychiatric care (45.5% vs. 14.3%, *p <* 0.001). In 10% of cases patients were unable to assure they would not act on their suicidal ideations/intentions, with a vast majority of these patients being hospitalized (18.5% vs. 0.1%, *p <* 0.001).

A table featuring all items of PPA assessed can be found in the supplementary data.

### Multivariate logistic regression analyses to determine predictors of admission to inpatient psychiatric care

#### Multivariate logistic regression analysis to determine sociodemographic and circumstantial predictors of admission to inpatient psychiatric care

The results of the multivariate logistic regressions are presented in Tables 4 and 5.

**Table 4:** Multivariate logistic regression analysis including sociodemographic and circumstantial variables associated with inpatient psychiatric admission

	OR	95% CI		*p*-Value
		LL	UL	
Gender	1.06	0.90	1.26	0.480
Age	1.01	1.00	1.01	0.100
Aggressive towards people	2.20	1.40	3.43	< 0.001**
Mechanical restraint	2.29	1.12	4.70	0.023*
Previous psychiatric treatment	1.88	1.54	2.29	< 0.001**
**Means of presentation (reference = walk-in)**				< 0.001**
By ambulance	1.48	1.23	1.80	< 0.001**
By police escort	2.18	1.54	3.11	< 0.001**
Transfer	6.20	3.17	12.14	< 0.001**
**Suicide attempt prior to presentation (reference = no suicide attempt)**				< 0.001**
Via intoxication	9.74	2.96	32.05	< 0.001**
Via self-injury	13.66	1.80	103.75	0.011*
Via other methods (e.g. strangulation, combination of methods)	25.30	3.36	190.81	0.002*
**Day of the week (reference = Sunday)**				0.022*
Tuesday	1.48	1.06	2.07	0.021*
Wednesday	1.49	1.07	2.07	0.017*
Retired (reference = (self-)employed)	1.89	1.29	2.78	0.001*
Living alone (reference = with others)	1.54	1.20	1.97	< 0.001**

OR: odds ratio, 95% CI: 95% confidence interval, LL: lower Limit, UL: upper limit, transfer: transfer from another hospital or another medical discipline within Hannover Medical School; * statistically significant, ** highly statistically significant

Omnibus test:* p* < 0.001, Nagelkerkes R-Square = 0.162, correctly predicted: 62.6%. Variables included in multivariate analysis after univariate testing: aggressive prior to presentation, aggressive towards people, aggressive towards objects, mechanical restraint, previous psychiatric treatment, means of presentation, intoxication, suicide attempt prior to presentation, day of the week, work status, living arrangement

**Table 5:** Multivariate logistic regression analysis including all variables associated with inpatient psychiatric admission

	OR	95% CI		*p*-Value
		LL	UL	
Gender	1.14	0.93	1.39	0.208
Age	1.01	1.00	1.02	0.003*
Aggressive towards people	2.85	1.70	4.75	< 0.001**
Previous psychiatric treatment	1.83	1.43	2.33	< 0.001**
Transfer (reference = walk-in)	6.26	3.02	12.98	< 0.001**
**Suicide attempt prior to presentation (reference = no suicide attempt)**				< 0.001**
Via intoxication	9.05	2.53	32.35	< 0.001**
Via self-injury	12.60	1.28	124.15	0.030*
Via other methods (e.g., strangulation. combination of methods)	17.36	1.93	156.01	0.011*
Wednesday (reference = Sunday)	1.55	1.04	2.31	0.030*
Living alone (reference = living with others)	1.55	1.16	2.08	0.003*
Unkempt/inappropriate attire	1.64	1.20	2.23	0.002*
Any disturbance of orientation	3.66	2.52	5.31	< 0.001**
Inhibited/retarded thinking	1.40	1.05	1.87	0.022*
Incoherence/derailment	1.75	1.09	2.81	0.021*
Delusions in general	2.25	1.64	3.10	< 0.001**
Acustic hallucinations	1.63	1.11	2.41	0.013*
Visual hallucinationens	2.89	1.64	5.10	< 0.001**
Hopelessness/despair	2.36	1.73	3.23	< 0.001**
Anxiety	1.89	1.13	3.15	0.015*
Inhibition/lack of drive	1.63	1.29	2.06	< 0.001**
Increased drive	1.68	1.11	2.53	0.013*
Motor restlessness/tensness	1.59	1.23	2.06	< 0.001**
Motor retardation	1.75	1.18	2.60	0.005*
Mannerisms/histrionics	2.35	1.15	4.80	0.019*
Suicidal ideation (reference = no suicidal ideation)	3.79	2.92	4.93	< 0.001*
**Suicidal intent (reference = distance to suicidal intent)**				< 0.001*
Lack of distance to suicidal intent	116.04	15.94	844.79	< 0.001*
Missing information	7.23	3.28	15.96	< 0.001*

OR: odds ratio, 95% CI: 95% confidence interval, LL: lower limit, UL: upper limit, transfer: transfer from another hospital or another medical discipline within the MHH; * statistically significant, ** highly statistically significant

In the first multivariate regression analysis including only sociodemographic and circumstantial variables (i.e., objective parameters, Table 4), SA prior to consultation in the PER had the largest influence on subsequent IPA: If presenting after a SA trough intoxication (OR 9.7, 95% CI 3.0-32.1), self-injury (OR 13.7, 95% CI 1.8-103.8) or other methods (e.g., strangulation, combination of methods; OR 25.3, 95% 3.4-190.8) compared to no SA, patients were admitted to inpatient psychiatric care significantly more often.

Patients arriving as a transfer from other hospitals or from another medical discipline within the MHH were six-times more likely to be admitted compared to patients having presented by walk-in (OR 6.2, 95% CI 3.2–12.1). Patients arriving by ambulance (OR 1.5, 95% CI 1.2–1.8) or in police escort (OR 2.2, 95% CI 1.5–3.1) also significantly predicted subsequent IPA.

Presenting at the PER on Tuesdays or Wednesdays was found to increase the odds of subsequent IPA by a 1.5-fold (95% CI 1.1–2.1).

If the patients had received previous psychiatric treatment, they had a 1.8-fold higher risk of IPA (95% CI 1.5–2.3). Patients with aggressive behavior towards other people prior to presentation (OR 2.3, 95% CI 1.1–4.7) and the use of mechanical restraint during the stay in the PER (OR 2.2, 95% CI 1.1–4.6) also showed an increased risk of IPA. Living alone compared to living with others increased the risk of IPA 1.5-fold (95% CI 1.2-2.0). Patients who were retired showed a 1.9-fold (CI 95%, 1.3–2.8) increased risk of IPA compared to patients who were (self-)employed.

#### Multivariate logistic regression analyses to determine sociodemographic, circumstantial and psychopathological predictors of admission to inpatient psychiatric care

Omnibus test *p <* 0.001, Nagelkerkes R-Square = 0.488, 77.6% correctly predicted. Variables included in multivariate analysis after univariate testing: age, gender, aggressive prior to the presentation, aggressive towards people, aggressive towards objects, mechanical restraint, previous psychiatric treatment, means of presentation, intoxication, suicide attempt prior to presentation, day of the week, work status, living arrangement, unkempt/inappropriate attire, disorders of consciousness, any disturbance of orientation, disturbed apperception/concentration, disturbed memory, inhibited/retarded thinking, accelerated thinking, circumstantial thinking, restricted thinking, rumination, rambling, tangential thinking, incoherence/derailment, delusions in general, delusions of reference, other delusions/delusional content, auditory hallucinations in general, hearing voices, visual hallucinations, any ego disturbance, suspiciousness, feeling of loss of feeling, hopelessness/despair, anxiety, dysphoria/irritability, inner restlessness, feelings of inadequacy, inhibition/lack of drive, increased drive, motor restlessness/tenseness, motor retardation, mannerisms/histrionics, social withdrawal, aggressiveness, self-harm, lack of insight into illness, circadian disturbances/disturbances of sleep and vigilance, consultation by other specialties, suicidal ideation, suicidal intent.

In the overall multivariate regression analysis (Table 5), suicidal ideation and suicidal intent were found to be the strongest predictor of IPA: Patients unable to assure they would not act on their suicidal ideation/intention were at least 15-times more likely to be admitted to inpatient psychiatric care (OR 116.0, 95% CI 15.9-844.8). Also, the lack of information regarding suicidal intention greatly correlated with IPA (OR 7.2, 95% CI 3.3–16.0). The presence of suicidal ideation increased the risk for IPA 3.8-fold (95% CI 2.9–4.9). Moreover, delusions (OR 2.3, 95% CI 1.6–3.1) showed a significant influence on IPA, as well as visual hallucinations (OR 2.9, 95% CI 1.6–5.1). Disturbance of orientation and feeling of hopelessness/despair were each found to significantly increase the risk of IPA by a 2.3 to 3.7-fold (95% CI 2.5–5.3 and 1.7–3.2). Patients presenting with inhibition/lack of drive were also more likely to be admitted to inpatient psychiatric care (OR 1.6, 95% CI 1.3–2.1).

The risk of IPA increased significantly with the patients age (OR 1.01, 95% CI 1.0-1.02).

Some of the sociodemographic and circumstantial variables with a significant influence in the first multivariate regression analysis lost significance in the overall multivariate regression analysis: Referral by ambulance and accompaniment by the police did not represent independent predictors any longer, as well as being retired. The same applies for consultations taking place on Tuesdays.

## Discussion

2580 patients sought consultation at MHH’s PER in the year 2022. The number of patients presenting at the MHH’s PER per year seem to be stable, as a similar number of consultations was found by Seifert et al. [27] in the year 2019/2020 and Ziegenbein et al. [26] in 2002 (mean 2607, SD 26). Out of the 2387 consultations included in the analysis, 1300 of the cases (54.5%) resulted in subsequent IPA, indicating a very similar admission rate as found by Ziegenbein et al. [26] at the same hospital twenty years prior.

Many previous studies analyzing inpatient admission after presentation in PERs have failed to create conclusive results since they considered small samples of only a few hundred patients [22, 23, 36, 37], used short observation periods of under one year [14, 18, 22, 23, 36, 37] or did not perform multivariate analysis [15, 22]. Additionally, most research relies on data from the last millennium [7, 10–15] and can be considered at least partly outdated. Furthermore, most studies include data derived in the USA, with only a few stemming from Europe [11, 16–18, 38] and even less in Germany specifically with only three studies [2, 25, 26]. Bassuk et al. [39] found significant differences between US-American and European emergency room patients regarding sociodemographic factors, clinical presentation and admission decision, emphasizing the importance of regional research in this matter. Therefore, in the following discussion, we decided only to include research with data from 2000 or later and only if included a multivariate analysis to determine risk factors of IPA.

To the best of our knowledge, this study is the first to extensively examine individual elements of the PPA in regard to inpatient psychiatric admission. Among the 53 variables that met the inclusion criteria for the multivariate logistic regression analysis, out of which 39 were aspects of PPA, 23 remained significant in the overall multivariate regression analysis as independent predictors of IPA. By including both objective (i.e., circumstantial and sociodemographic) and the more subjective psychopathological factors in one regression model, we were able to demonstrate how these factors significantly interact with one another during the process of the admission decision. In doing so, risk factors for IPA as reported by other researchers who did not extensively include psychopathology when determining risk factors for IPA were not significant in the present study. Thereby, the present study found several aspects of PPA to be the most significant predictors of IPA: suicidal ideation, suicidal intent, prior SA, prior aggressive behavior against people, previous psychiatric treatment, transfer as means of presentation, disturbance of orientation, delusions, visual hallucinations, hopelessness/despair, lack of drive and motor restlessness/tenseness.

### Sociodemographic and circumstantial variables predicting admission to inpatient psychiatric care

Among the sociodemographic and circumstantial variables, transfer (i.e., from a different hospital or from another medical discipline within MHH) as means of presentation was an important predictor for IPA in the present study. In general, it can be assumed that patients are transferred to MHH’s department of psychiatry or within the MHH with the intent of subsequent IPA. In most cases, the physicians initiating the transfer have already consulted a psychiatrist (via phone or liaison psychiatry) who indicated the need for IPA. No other study was found to have examined this mode of referral.

This study also found referral by ambulance and police to increase the risk of IPA in the sociodemographic/circumstantial multivariate regression analysis, as found by Bahji et al. [40]. However, referral by ambulance and police lose their significance in the overall multivariate regression analysis, in which aspects of PPA are also included. This is most likely because referral by ambulance and police escort are not independent risk factors but co-occur with certain variables of PPA which in fact increase risk of IPA according to this study, e.g., aggressive behavior, formal thought disorder, disorders of thought content and affect disturbance. These variables also coincide with a higher likeliness for police involvement [41, 42]. Nonetheless, referral by ambulance and police may function as important indicators of subsequent IPA prior to the examination by the psychiatrist on call.

Previous psychiatric treatment, including *inpatient and outpatient care*, was also found to be a significant predictor of IPA in another study [2]. On the other hand, Unick et al. [21] could not find a significant influence of previous *inpatient* admission. This discrepancy may indicate former outpatient psychiatric treatment to have an meaningful impact on IPA since it was included in the present and Kirchner et al. [2]’s study where significance could be found. This may suggest that inpatient psychiatric care is required, when outpatient care has proven insufficient. However, previous psychiatric treatment increased the odds (i.e., OR 1.8) for subsequent IPA to a lesser extent than most other risk factors detected in the present study, again suggesting that perhaps other factors are more decisive, especially aspects of PPA.

This study found living alone in comparison to living with others to slightly (i.e., OR 1.54) but significantly increase the risk of IPA. The impact of a patient’s living arrangements on the admission decision can be manifold and may serve as an indicator for (perceived) social support. In so, patients living alone and therefore with perhaps lacking social support may preferentially be admitted to inpatient psychiatric care than those living with others. On the other hand, serious mental illness often correlates with lack of social support and/or relationships [43] suggesting that these variables may reciprocally influence each other. Additionally, the disruption of the social network (e.g., separation from family members or spouses) can aggravate the psycho-pathological impairment [38], for example by worsening symptoms of anxiety.

The relevance of sex as a predictor of IPA is somewhat debatable. Sex-specific differences regarding psychiatric pathology have been described by other authors, e.g., higher rates of SA among females [44], but higher rates of suicides among males [45]. While a few studies found female sex to correlate with higher risk of admission [21, 26], most other studies could not find a significant influence of sex [2, 19, 25, 38, 40]. The present study included the variable gender as opposed to biological sex. Gender was not significantly associated with IPA, most likely because of the inclusion of a large number of psychopathological variables which more adequately necessitate the need for IPA.

Increasing age further significantly predicted IPA in the present study. Per year in age, the risk of subsequent IPA increased by 1%. Hamilton et al. [19] and Unick et al. [21] also found higher age to be a significant predictor for IPA, whereas Martin-Santos et al. [38] found that patients under the age of 45 years had an increased risk of IPA in comparison to patients over 45 years old. Several other studies did not detect a significant influence of age [2, 25, 40]. One possible explanation for these contradicting results regarding age may be confounded by other variables that were included in these studies: In so, two studies that considered self-care ability in the multivariate analysis, did not find increasing age to increase risk of IPA [25, 38]. These finding suggests that self-care may more adequately predict IPA than age itself, as this ability often deteriorates (e.g., as a result of dementia or immobility) with age [46, 47].

### Aspects of psychopathological assessment predicting admission to inpatient psychiatric care

This study did not include the main diagnosis in the multivariate logistic model since diagnoses in the PER function as provisional working diagnosis and were found to be unreliable [34, 35]. Additionally, the presence of a certain diagnosis may not adequately represent the patient’s current emotional state. Examination of the PPA and therefore the foundation of the diagnosis may provide a more fundamental understanding of the decision-making process regarding IPA, as it is not the diagnosis per se that determines the necessity of treatment, but the symptoms the patient is currently suffering from. Additionally, when analyzing the frequencies of diagnoses, one must bear in mind the bidirectional influences of many diagnoses. In so, it is often difficult to determine which diagnosis is indeed the “primary diagnosis”. For example, a depression can lead to alcohol abuse and addiction, which can worsen symptoms of depression. Another reason for the exclusion is the limited comparability to other studies since different classification systems are used (e.g., ICD-10 and DSM-V).

Current research on the decision of IPA after emergency psychiatric presentation is characterized by low consistency, partly due to variations regarding included variables and their definitions. As one of the few congruent research results, suicidal ideation and suicidal behavior (SB) was often found to be an independent predictor of IPA [19, 25, 26, 38]. Consistent with these findings, suicidal intent was identified as the strongest predictor of IPA among the PPA variables examined in this study. Unsurprisingly, lack of distance to suicidal intentions and missing information on this item increased the probability of IPA significantly as the consequence of not admitting patients with acute suicidal intentions may undeniably be fatal. Similarly to Michaud et al.’s findings, the level of suicidal intent significantly correlated with the risk of IPA [48]. The same applies for the preliminary stage, namely suicidal ideation: Even though the presence of suicidal ideation present a smaller risk of suicide than suicidal intent, it may still lead to suicide if not addressed adequately [49].

This study found SA prior to consultation to be an independent risk factor, contrary to Ziegenbein et al. [26]’s study. Our findings are not surprising, as literature suggests previous SA to present a major risk factor for subsequent suicide or SA [50, 51] which physicians may attempt to deter by the IPA. Since the confidence intervals of the different methods of SAs are especially wide (Table 5), the strength of this predictors cannot be evaluated appropriately, but the existence of an influence seems apparent. However, Michaud et al.’s found serious SAs, “defined by the fact that [they] required hospitalization for more than 24 h and met one of the following criteria: management in a specialized unit (e.g., intensive care), surgery under anaesthesia, or prescription of major medications” [48] to be independent predictors of IPA [48]. It is likely that a majority of patients who meet these criteria were not included in this study, since they were not treated primarily by the psychiatrist on call but e.g., by surgical disciplines. This may explain why a distinction between different modes of SA and their relative impact on IPA was inconclusive in our findings.

The higher risk of IPA in presence of disorder of orientation may be explained by it being the most fundamental psycho-pathological feature after impairment of consciousness. Any of such disturbance may interfere with the patient’s decision-making abilities and therefore lead to self-endangerment [52]. Moreover, impaired sense of orientation may be caused by potentially life-threatening psychiatric emergencies such as alcohol withdrawal and delirium [5]. Visual hallucinations also present a hallmark symptom of delirium [5] explaining their significant impact on IPA.

Delusions may lead to self-endangerment or endangerment of others as a result of misinterpretation of reality. For example, the presence of delusions was found to be a risk factor for violent behavior in patients with schizophrenia [53], thus providing a possible explanation for increased risk of IPA in presence of delusions. Additionally, delusions in patients with depression present an indication for a more severe course of illness [54].

Being hopeless/desperate and the inhibition/lack of drive each influence the admission decision possibly because of the association with high subjective burden and loss of functionality [55].

However, analyzing the PPA also has possible obstacles. The PPA is the result of direct questioning by the physician (e.g., “how would you describe your current mood?“), observations made by the physician (e.g., how the patient’s mood appears to be, formal thought disorders) and information the patient spontaneously offers. This process is of course somewhat subjective, despite efforts to objectify the PPA by using an established documentation system such as the AMDP-System. Further, certain circumstantial and sociodemographic information (e.g., substance use, police escort) may lead to a bias in the evaluation of aspects of PPA. Similarly, the patient’s potential unwillingness to disclose of certain aspect (e.g., substance use) may have resulted in an under-reporting of these items and a possible bias. For example, Elangovan et al. [56] found the failure to identify cocaine abuse can lead to unnecessary hospitalization and inappropriate management because of misjudgment of symptom severity.

### Impact of frequent visitors on sociodemographic, circumstantial and psychopathological predictors of inpatient psychiatric admission

A small but relevant percentage of patients are frequent visitors. Although no standardized definition present, researchers agree on their great impact on PERs for these patients claim a relatively high amount of already limited resources [6, 31, 57, 58]. In the present study, frequent visitors (6.1% of all patients) made up for 27.5% of the cases, confirming the previously stated impact on the PER’s resources. The present study considered each presentation separately, regardless of the patient being a frequent visitor or not. This may have resulted in an over-representation of certain items associated with frequent visitors such as substance abuse, unemployment and prior psychiatric hospitalization [6]. For this group of patients, examining the impact of PPA items on the admission decision is particularly important: The analysis of the PPA helps to differentiate between different consultations of the same patient and allows to identify the reason for IPA. Without the information of the PPA seemingly similar cases (i.e., same patient and therefore same sociodemographic variables and diagnoses) would present with different admission outcomes without a possible explanation.

### Limitations

The results presented in this study should be interpreted in the context of its limitations.

First, as the data was collected from a single institution over the course of one year, the findings from this study may not apply to other settings or time periods. For example, the distribution of the main diagnoses found in patients presenting to a PER in Switzerland strongly deviated from our findings. We found alcohol-related disorders to be the most frequent main diagnosis (23.9%), whereas Costanza et al. only found 10% of patients to present with a primary diagnosis of substance use disorders [59]. Secondly, as a result of the retrospective approach of extracting data out from already existing documentations only information found in the respective documents was available for each case. However, by using this approach, the routine processes in the PER were not interfered with, therefore enabling an authentic, real-life insight. The present study only includes patients presenting in the PER. That means cases in which another discipline was the treatment provider are largely not included in the present study, unless the patient was triaged to psychiatry after completion of somatic care. This may lead to under-representation of specific psychiatric patients, for example of patients with severe SAs with consecutive indication of somatic treatment. Also, the present study does not consider the legal status of admission (i.e., voluntary vs. compulsory admission). It can be assumed, that patients admitted against their will may be more severely ill than those admitted voluntarily. Moreover, due to the largely unstructured character of the psychiatric interview, items (e.g., certain aspects of PPA, employment status) were often missing. On the other hand, it can be assumed that information pertinent to the emergency situation are almost always investigated and documented (e.g., suicidal ideation, aggressive behavior). These deficits in documentation could be evaded in the future by using a standardized questionnaire, however, their use is uncommon in the emergency setting with only a minority of about 5% of the PERs in Germany using such standardized questionnaires [1]. The dichotomous documentation in our study leads to another limitation: Since many items are only extracted as given or not given, severity of symptoms (e.g., severe psychomotor restlessness) influencing IPA cannot be evaluated. To counteract this concern, some important items were graded or separated for a more nuanced evaluation (e.g., “suicidality” separated in “suicidal ideation”, “suicidal intent” and “SA prior to presentation”). Finally, the present study was unable to consider all possible factors influencing IPA, e.g., bed availability, which has been described as a main predictor of voluntary admission by others [60]. Therefore, all results presented in this study have to be considered within the limited borders of its exploratory design and no single variable can be *proven* to be an independent risk factor for IPA, as possible confounders cannot be excluded with certainty.

## Conclusion and clinical implication

The decision whether a patient is admitted to inpatient psychiatric care is influenced by clinical, sociodemographic and circumstantial variables as well as their interactions. The present study detected several variables that were significantly and relevantly associated with the risk of IPA. Aggressive behavior towards other people and SA prior to the consultation, as well as orientation disorders, visual hallucinations, delusions, affective impairments, inhibition/lack of drive, suicidal ideation and suicidal intent appear to be of high predictive value in deciding which patients require IPA. Of course, this does not imply that other aspects of the PPA should be neglected.

Many aspects, such as the distribution of the main diagnoses (see above), demographics (e.g., gender, age, work status [39]) and admission rates [48], may be subject to regional differences. Therefore, a precise analysis of specific aspects of PPA using a well-established assessment system such as AMDP as performed in the present study may be helpful to determine more objective risk factors for subsequent IPA. Since this study included a particularly large number of such aspects, the resulting findings may be more applicable to other populations. Of course, further research and ideally meta-analyses are necessary to further elucidate this question.

IPA of a patient after emergency presentation indicates that less extensive, outpatient-based care is currently insufficient. Understanding which patients require inpatient care and which circumstances contribute to the inpatient admission, is the basis for developing more specialized ambulatory care. This could reduce the need for IPA in the future.

Lastly, extracting predictors of inpatient admission can be used for the implementation of guidelines serving substantiated decision making and therefore improve psychiatric care.

### Electronic supplementary material

Below is the link to the electronic supplementary material.

**Additional file 1: “PPA Table”**. All aspects of the psychopathological assessment (PPA) assessed as found in the study population. IPA: inpatient psychiatric admission, n: number of consultations, df: degrees of freedom. * statistically significant, ** highly statistically significant

## Data Availability

The data can be made available by the corresponding author upon reasonable request.

## List of abbreviations

95% CI: 95% confidence interval
AMDP: Arbeitsgemeinschaft für Methodik und Dokumentation in der Psychiatrie
df: Degrees of freedom
IPA: Inpatient psychiatric admission
LL: Lower limit
MHH: Medizinische Hochschule Hannover (Hannover Medical School)
n: Number of consultations
OR: Odds ratio
pats.: Patients
PER: Psychiatric emergency room
PPA: Psychopathological assessment
SA: Suicide attempt
SB: Suicidal behavior
UL: Upper limit
y/o: Years old
